# Prevalence of intestinal parasites and associated risk factors in HIV positive and negative patients in Northwest Region, Cameroon

**DOI:** 10.1038/s41598-022-20570-4

**Published:** 2022-10-06

**Authors:** Ngum Helen Ntonifor, Abongwe Sidney Warra Tamufor, Lem Edith Abongwa

**Affiliations:** 1 Department of Biological Sciences, Faculty of Science, The University of Bamenda, Bambili, Cameroon; 2 Department of Zoology and Animal Physiology, University of Buea, Buea, South West Region Cameroon; 3 African Centre of Excellence for Genomics of Infectious Diseases (ACEGID), Redeemer’s University, Ede, Osun State Nigeria

**Keywords:** Microbiology, Medical research, Molecular medicine

## Abstract

Epidemiological understanding of intestinal parasitic infections is essential for the effective management of HIV infection. Therefore, this study was designed to assess the burden of intestinal parasites and associated risk factors. A cross-sectional study was conducted from May to December 2020 during which 200 HIV positive and 200 HIV negative participants were recruited. A total of 400 stool and venous blood samples were collected and used to identify the different intestinal parasites and for HIV diagnosis and viral load determination respectively. Results obtained revealed that the overall prevalence of intestinal parasites was 11% (44/400). Intestinal parasitosis was significantly (*p* = 0.025) higher in HIV-positive individuals 14.5% (29/200). Similarly, the prevalence of multiple parasitic infection 4.5% (18/400) and opportunistic helminths 3% (6/400) were insignificantly (*p* > 0.05) higher among HIV-positive individuals. Furthermore, prevalence of intestinal parasites was significantly (*p* = 0.004) greater in patients with viral load of > 1000 copies/mL 24.3% (13/46). Age group > 65 years, self-employment, living in Sub-urban areas, being HIV positive, primary level of education, use of potable tap water, and the use of water system toilets for faeces disposal were identified as associated risk factors to intestinal parasites. Intestinal parasites remain public health concern among patients with HIV. Prompt and effective antiretroviral treatment is required to reduce the intensity of the parasite.

## Introduction

Intestinal parasitic infection is a condition in which parasites infect the gastrointestinal tract of humans^1^. The presence of these parasites can damage or sicken the host even though in most cases it is self-limiting except in immunocompromised individuals such as persons with Acquired Immune Deficiency Syndrome (AIDS) or Cancer^2^. The infection rate of intestinal parasites is remarkably high in Sub-Saharan Africa which harbors the highest prevalence of HIV positive patients, accounting for more than 70% of the global burden of infection thus indicating that, the burden of the intestinal parasite is affected by the immunological state of the patient^1^. Reports from previous studies in different regions in Cameroon revealed that the prevalence of at least one intestinal parasite ranges from 21.92 to 34.5%^3,4^. Furthermore, studies on HIV and intestinal parasitic co-infection in other towns gave a prevalence range of 14.4 to 57.4%^5,6^. Intestinal parasites and HIV co-infections are some of the neglected areas in HIV research in Cameroon although HIV generally has become a major public health concern in Cameroon and beyond. Even though the concerns regarding opportunistic parasitic infections among HIV positives have been widely recognized, only a few relevant investigations have been reported in Cameroon to establish the presence of intestinal parasites in this susceptible group. However, no study has been carried out in Tubah Health District which is a subdivision in Bamenda with an HIV prevalence of about 1.25%^7^. It was therefore important that such a study be carried out to determine the prevalence of intestinal parasites and associated risk factors in HIV positive and negative patients at Tubah, Northwest region of Cameroon.

## Material and methods

### Study design

The study was a cross-sectional, hospital-based study from May to December 2020. HIV-positive patients coming for drug refill and HIV-negative individuals visiting Tubah District Hospital for consultation for other health issues other than HIV drug refill were recruited into the study after signing the informed consent form. A structured, pre-tested questionnaire was used to collect data on socio-demographic characteristics and biodata on knowledge, attitude, and practices on helminths. Stool samples (loose and watery, semi-formed and hard) were collected once for parasitic analysis and venous blood samples for HIV diagnosis and viral load measurement. Data collected were analyzed using SPSS vs 26 and the results were presented using tables and figures.

### Ethical approval

Ethical clearance was obtained from the Bamenda University Ethical Review Board (2020/0249H/UBa/IRB) and authorization from the Director of the Tubah District Hospital. An information sheet was used to explain the objectives, procedure, benefits, and risks of the study. Adult informed consent from patients aged ≥ 18 years and minor assent with parental permission from patients aged < 18 years were received from participants before inclusion in the study. The confidentiality of each participant was ensured by using codes.

### Study area and population

**Figure 1:**
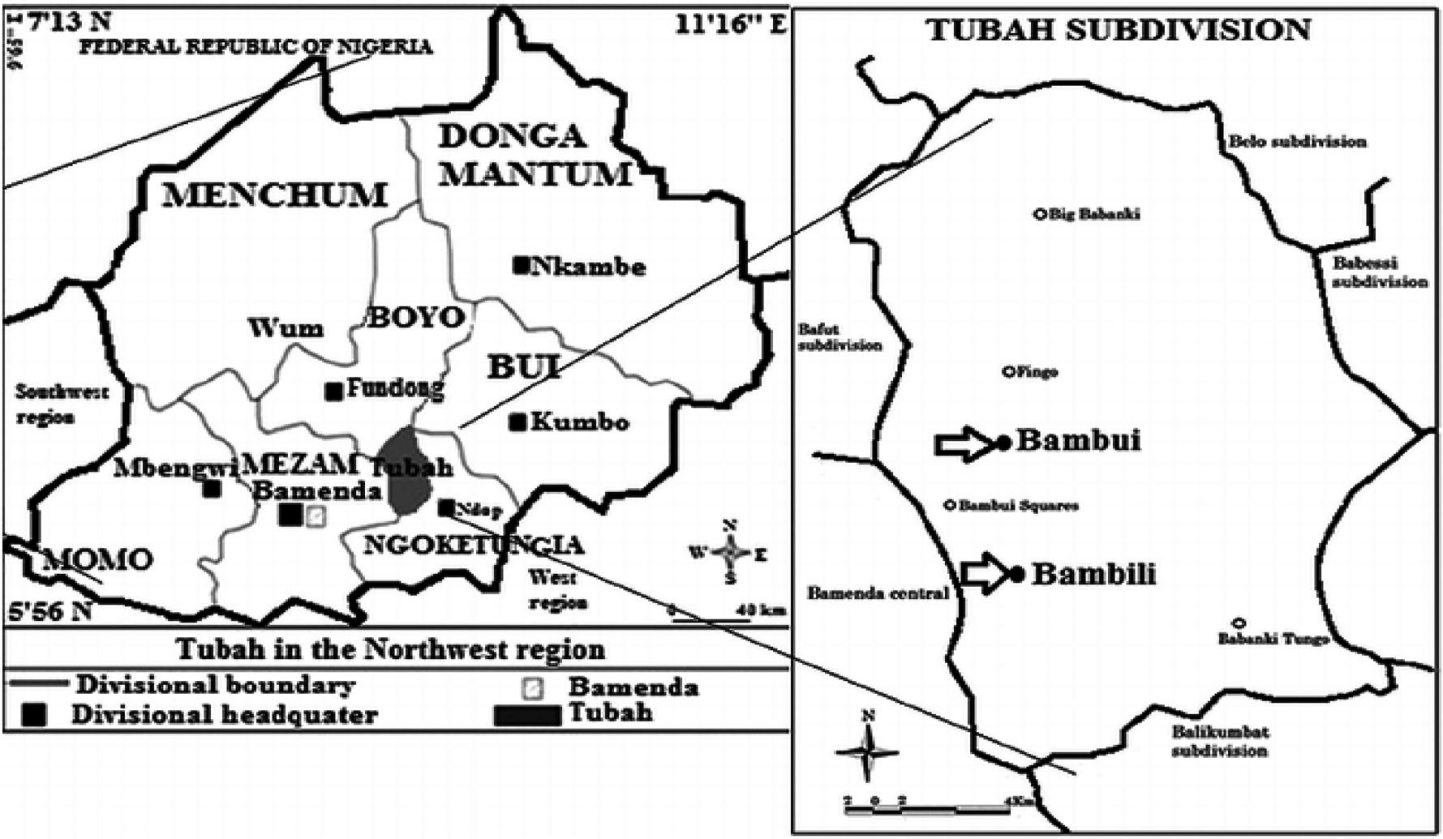
Map of Bamenda showing the study area.

This study was carried out at the Tubah District Hospital Bambui, in the Tubah subdivision, located close to the major city of Bamenda town (Fig. 1). This hospital act as a reference hospital to other rural communities like Bambili, Babanki-Finge, Baforkum, Bafut, and Nkwen. This site was chosen because it is a rural area with an HIV treatment center where no such study has been carried out and acts as a reference hospital to the neighboring towns.

Inclusion criteria included: consented HIV positive patients on treatment for at least 6 months or HIV negative clients, participants who provide stool samples, and those living within the study area. Those on antihelminthics or any treatment for intestinal parasitism in the last two months preceding specimen collection, clients who didn't give their consent, and clients who did not provide the stool sample were excluded from the study.

### Sampling technique

The participants were first stratified into HIV positive and HIV negative. Participant selection from each group was done using the first come first serve technique.

### Sample size calculation

The study involved 200 HIV positive and 200 HIV negative individuals visiting the Tubah district hospital irrespective of their age, gender, race, occupation, or religion and who gave their consent. The minimum estimated size was calculated to be 91 HIV positive and 91 HIV negative using the Lorenz formula below^8^;

$${\text{N}} = \frac{{(Z_{1}^{2} - \alpha )P\left( {1 - P} \right)}}{{i^{2} }}$$

where Z_1_ − $\alpha$ = the normal distribution value = 1.96, P = Relative prevalence of HIV in the region = 6.3%^8^, i = precision (sampling error) = 0.05

$$N = \frac{{\left( {1.96} \right)^{2} x\left( {0.063} \right)\left( {1 - 0.063} \right)}}{{\left( {0.05} \right)^{2} }} = 91.$$

### Process of the survey

A pretested, close-ended questionnaire was administered to consenting participants to collect data on (age, sex, level of education, occupation, etc.), personal daily habits (living conditions such as the washing of hands, type of toilet, etc.), ART status (history of antiretroviral treatment), WHO HIV stage classification, information on compounding situations like the use of antihelminthics drugs and questions on the transmission and prevention method of helminthic parasites. Those who could read and write completed the questionnaires by themselves and those who couldn't read and write were assisted by the researcher. Assessment of the level of knowledge on the transmission and prevention of intestinal parasites was done by assigning 1 for any right answer and 0 for the wrong answer. The total score was 13 and a score range of 1–6 was considered poor while a score of 7–13 was considered good. As regards viral load, HIV patients with viral load readings ≤ 1000 copies/mL were considered viral suppressed while viral load readings > 1000 copies/mL were considered unsuppressed^9^.

### Sample collection and processing

For formed stool samples, participants were instructed to pass out stool on plastic foil paper and collect the part of the faeces containing mucus or blood (if present) in labelled clean, dry, and leak-proof containers. For watery stool, participants were told to collect about 10 mL of the stool as indicated on the container. Exactly 4 mL of venous blood was collected into EDTA tubes by a trained laboratory technician to test for HIV status in individuals with unknown status. While for HIV-positive patients, the blood sample was packaged using the triple packaging procedure, and transported to the Bamenda Regional Hospital Tuberculosis Reference Laboratory in a cooling box for viral load measurement.

## Laboratory analysis

### Preparation of wet mounts of stool for direct microscopy

A small portion of the freshly collected stool sample, (accepted only when the duration between collection and examination is less than or equal to 3 hours) was emulsified with 50µL of physiologic saline (0.85% NaC1 solution) on a clean glass slide. The preparation was covered with a coverslip and examined using the X10 and the X40 objectives of a light microscope (Olympus, Japan). To facilitate the identification of parasite ova, cysts, or trophozoites, another slide of stool emulsified with Lugol's iodine was prepared and covered with a coverslip to stain and differentiate the parasite cytoplasm^10^. An attempt was made to go through all the prepared fields before a sample was reported negative for parasites.

### Formol-ether concentration technique

This technique was carried out as described by Cheesbrough^11^. Briefly about 0.2 g of faeces were emulsified in 3 mLs of 10% formol water. Exactly 4 mLs of formol water was added to the preparation and mixed. The emulsified preparation was sieved and the filtrate was collected in a beaker and transferred to a centrifuge tube. Precisely 3 mL of ethyl acetate was then added to the preparation. The tube was stoppered and its content was mixed for a minute by gently inverting the stoppered tube and returning it to its upright position. The stopper was gently removed and the preparation centrifuged using a KA-1000 centrifuge at 3000 rpm for 5 min. Using a stick, the layer of faecal debris from the side of the tube was gently loosened and the tube was rapidly inverted to discard the supernatant, consisting of fecal debris, formol, and ether. The tube was then reverted to its upright position and allowed for the sediments to return to the bottom. To re-suspend the ova, the tube was gently taped and its content transferred to two slides, one of the slides was covered with a coverslip and observed using the X10 and X40 objectives. A drop of iodine was run under one of the slides to increase the visibility of parasite ova.

### Modified Ziehl Neelsen staining technique

The modified Ziehl Neelsen staining technique was carried out as described by Sandman et al.^10^. On the second slide, a smear was made on a clean glass slide and allowed to air dry. The preparation was fixed with absolute methanol. The slide was then stained with Carbol fuchsine for 10 min, followed by rinsing of the stained slides with water. The preparation was decolorized with acid alcohol (99 mL of 96% alcohol and 1 mL hydrochloric acid), followed by rinsing in tap water and counterstaining in methylene blue for one minute. The slide was then rinsed, dried, and observed using the oil immersion objective of an Olympus microscope.

### Rapid HIV test (Alere Determine™ HIV–1/2 Ag/Ab Combo)

HIV was determined as described by the manufacturer's procedure^12^. Briefly, 50 μL of whole blood sample was applied to the sample pad (marked by the arrow symbol). A drop of chase buffer was added onto the sample pad and the results read between 20 and 30 min. The test was reported positive for HIV if a pink/red line appeared in the control area (C) and the lower test area (T) of the test strip and negative for HIV if a pink/red control line appeared only in the control area of the test strip.

### Confirmatory test (HIV-1/2 rapid testing by OraQuick^®^ ADVANCE)

A blood-filled collection loop was immersed into a developer solution vial and stirred. A divided pouch was inserted into the developer solution vial containing the specimen. Results were read after at least between 20 and 40 min in a well-lighted area. For a negative result, only the control (C) area showed a red line, while for a positive result two red lines appear in both the control (C) and the test (T) areas as described in the manufacturer's procedure^13^.

### Measurement of viral load

Samples were collected and transported to the Bamenda Regional Reference Hospital Tuberculosis Laboratory for analysis using the Abbott m2000SP real-time protocol (The Abbott RealTime HIV-1 assay that uses RT-PCR26 to generate amplified product from the RNA (ribonucleic acid) genome of HIV-1 in clinical specimens as described by Erba et al.^14^. Briefly, an RNA sequence that is unrelated to the HIV-1 target sequence was introduced into each specimen at the beginning of sample preparation. This unrelated RNA sequence is simultaneously amplified by RT-PCR and serves as an internal control (IC) to demonstrate that the process has proceeded correctly for each sample. The amount of HIV-1 target sequence that is present at each amplification cycle is measured through the use of fluorescent-labeled oligonucleotide probes on the Abbott m2000rt instrument. The amplification cycle at which fluorescent signal is detected by the Abbott m2000rt is proportional to the log of the HIV-1 RNA concentration present in the original sample.

### Statistical analysis and management

Data collected were entered into the computer using Microsoft Excel 16 and analyzed using SPSS vs 26 (Chicago, Illinois, USA). Different characteristics of study participants were described using mean, range, and percentage as appropriate. Univariate analyses were based on Pearson's Chi-square test to test the statistical significance of proportions. Fisher's exact test for contingency tables was used to test for significance in proportions of categorical data when the expected value was less than 5. Bivariate and multivariate logistic regression analyses were used to identify the risk factors. Odds ratios (OR) with a 95% confidence interval (CI) were used to measure the strength of associations between outcome and its correlates. All tests were two-tailed and statistical significance was defined as *p* < 0.05.

### Ethics approval and consent to participate

All methods used in this study were carried out following relevant guidelines and regulations. All experimental protocols were approved by his study protocol and were reviewed and approved by the Bamenda University Ethical Review Board (Project ID: 2020/0249H/UBa/IRB). Written informed consent was obtained from all subjects and/or their legal guardian(s).

## Results

### Characteristics of the study population

A total of 400 individuals participated in the study, 200 of these participants were HIV negative while 200 were HIV positive. The ages of participants ranged from 1 to 84 years with a mean ± standard error age of 34.87 ± 0.90 years and participants within the age group 21–35 years constituted the highest proportion 30.7% (123/400). More than half of the study population were females 62.3% (249/400), had attained a primary level of education 64.7% (259/400), came from suburban areas 76.3% (305/400), lived in homes with at least 1–4 persons 52.3% (209/400), and were self-employed 63.2% (253/400) carrying out activities such as farming, bike riding, trading, building, driving, etc. (Table 1).

**Table 1:** Socio-demographic distribution of the population.

Variables	Category	Frequency	Percentage (%)
Age group (years)	≤ 20	85	21.3
	21–35	123	30.7
	36–50	118	29.5
	51–65	56	14.0
	> 65	18	4.5
Gender	Males	151	37.7
	Females	249	62.3
Level of education	None	7	1.8
	Primary	259	64.7
	Secondary	70	17.5
	Tertiary	64	16.0
Occupation	Civil servant	5	1.3
	Self-employed	253	63.2
	Unemployed	142	35.5
Localities	Sub-urban	305	76.3
	Rural	95	23.7
Number of persons in a house	1–4	209	52.3
	5–9	178	44.4
	10–14	13	3.3

### Prevalence of different intestinal parasites and multiple parasitic infections among HIV positive and negative subjects

**Figure 2:**
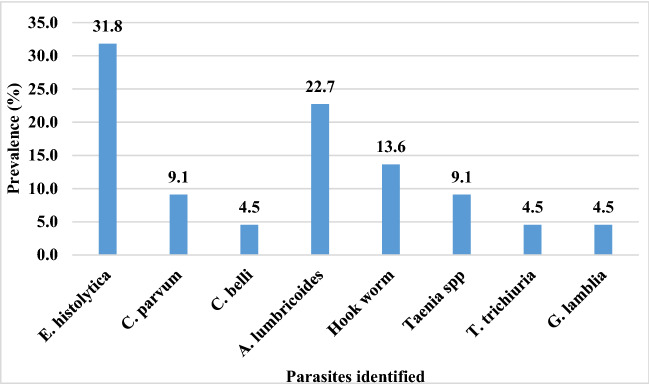
The prevalence of the different parasites identified among the study population.

**Figure 3:**
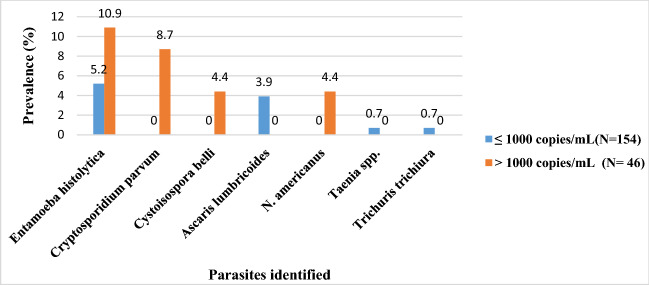
Comparison of parasites prevalence with viral load.

Results obtained revealed that the overall prevalence of intestinal parasites was 11% (44/400). The parasites identified were *Entamoeba histolytica, Cryptosporadium parvum*, *Cystoisospora belli*, *Gardia intestinalis, Ascaris lumbricoides*, Hookworm spp, *Taenia* spp, and *Trichuris trichiura* (Fig. 2). The most prevalent parasite was *Entamoeba histolytica* 22.7% (10/44) followed by *Ascaris lumbricoides* with 31.8% (14/44). Intestinal parasitosis was significantly (*p* = 0.025) higher in HIV positive individuals 14.5% (29/200) than HIV negative individuals 7.5% (15/200) (Table 2). Similarly, the prevalence of multiple parasitic infections 4.5% (18/400) and opportunistic infections 3% (6/400) was insignificantly (*p* = 0.076) higher among HIV-positive individuals. The viral load ranges from target not detected (TND) to 61.221 copies/mL. Prevalence of intestinal parasites was significantly (*p* = 0.004) greater in patients with viral load of > 1000 copies/mL 24.3% (13/46) compared to patients with viral load ≤ 1000 copies/mL 10.4% (16/154) (Fig. 3).

**Table 2:** Prevalence of different intestinal parasites and parasites multiple infections among HIV positive and negative subjects.

Variables	HIV status				χ^2^	*p* value
	Positive (%)		Negative (%)			
	N^o^ examined	N^o^ infected (%)	N^o^ examined	N^o^ infected (%)		
	200	29 (14.5)	200	15 (7.5)	5.005	0.025
**Protozoa**						
*E. histolytica*	200	13 (6.5)	200	1 (0.5)	14.115	0.003
*C. parvum*	200	4 (2.0)	200	0 (0.0)		
*C. belli*	200	2 (1.0)	200	0 (0.0)		
*G. intestinalis*	200	0 (0.0)	200	2 (1.0)		
**Total**	200	19 (9.5)	200	3 (1.5)		
**Helminths**						
*A. lumbricoides*	200	6 (3.0)	200	4 (2.0)	1.901	0.593
*Hookworm*	200	2 (1.0)	200	4 (2.0)		
*Taenia* spp.	200	1 (0.5)	200	3 (1.5)		
*T. trichiuria*	200	1 (0.5)	200	1 (0.5)		
**Total**	200	10 (5.0)	200	12 (6.0)		
**Multiple infections**						
*E. histolytica* + *C. parvum*	200	4 (2.0)	200	0 (0.0)	8.454	0.076
*E. histolytica* + *A. lumbricoides*	200	4 (2.0)	200	1 (0.5)		
*E. histolytica* + *N. americanus*	200	2 (1.0)	200	0 (0.0)		
*A. lumbricoides* + *N. americanus*	200	1 (0.5)	200	3 (1.5)		
*A. lumbricoides* + *Taenia* spp.	200	2 (1.0)	200	1 (0.5)		
**Total**	200	13 (6.5)	200	5 (2.5)

### Assessing risk factors associated with any of the intestinal parasites

Bivariate regression analyses showed that parasitic infections were significantly (*p* < 0.05) high in the age group > 65 years (cOR: 6.152, 95% CI 1.563–24.251), occupation (Self-employed, [cOR: 3.304, 95% CI 1.432–7.621], Unemployed [cOR: 0.052, 95% CI: 0.452–1.251]), patients from Sub-urban (cOR: 2.004, 95% CI 1.032–3.889), HIV positive patients, those who had attended primary education (cOR: 9.844, 95% CI 1.322–73.27), who use portable tap water (cOR: 3.145, 95% CI 1.552–6.371), and water system toilet(cOR: 2.316, 95% CI 1.174–4.566). The prevalence of parasitic infection among those participants of the age groups 21-25 years (*p* = 0.07), 36-50 years (*p* = 0.08), 51-65 years (*p* = 0.09), and those who had attended secondary level of education (*p* = 0.07) only showed a trend. However, gender, number of occupants per house, type of house, and Keeping of animals or pets were not predictive (*p* > 0.1) for the presence of any of the parasites (Table 3). Variables (age, occupation, location, HIV status, level of education, and the use of portable water) that showed significance of *p* < 0.05 or a trend of *p* = 0.05–0.09 in the bivariate analysis were used for the multivariate analysis. Although patients > 65 years were at risk of being infected, the risk was lower by 3.16 (1.51–23.95) after controlling for the other variables. Self-employed was also found to be a significant predictor of parasitic infection in this study, and the risk of having any parasitic infection in patients increased to 2.76 (95% CI = 1.103–6.892) after controlling for the other variables. Similarly, the risk increased in those who had HIV and those living in Sub-urban towns in the multivariate analysis. Furthermore, persons who had attended primary education had a lower risk 8.48) of being infected with any of the parasitic infections (AOR = 8.48, 95% CI = 1.13–63.62) after controlling for the other variables. Our data reveal that participants who used portable tap water and water system toilet had a higher risk (3.26 and 2.35 respectively) of being infected. Even though participants of the age groups 21-25 years (*p* = 0.07), 36–50 years (*p* = 0.08), 51-65 years (*p* = 0.09), and those who had attended secondary level of education (*p* = 0.07) showed a trend in bivariate analysis indicating an insignificant higher risk of parasitic infection, these factors were not predictors of any of the parasites as in Table 3 below.

**Table 3:** Risk factors associated with the prevalence of intestinal parasites in the study area.

Variables	Prevalence		Bivariate analysis		Multivariate analysis	
	N^o^ examined	N^o^ infected (%)	cOR (95% CI)	*p* value	aOR (95%CI)	*p* value
**Age group (years)**						
≤ 20	85	5 (5.9)	a	a	a	a
21–35	123	14 (11.4)	2.995 (0.928–9.667)	0.07	3.064 (1.180–13.641)	0.24
36–50	118	14 (11.9)	2.857 (0.884–9.230)	0.08	1.671 (1.234–15.085)	0.48
51–65	56	6 (10.7)	3.205 (0.844–12.173)	0.09	2.820 (1.084–17.263)	0.13
> 65	18	5 (27.8)	6.152 (1.563–24.251)	0.01	3.158 (1.512–23.950)	0.04
**Gender**						
Males	151	16 (11.2)	1.07 (0.558–2.049)	0.84	1.01 (0.521–1.942)	0.99
Females	249	28 (10.6)	a	a	a	a
**Occupation**						
Self-employed	253	37 (14.6)	3.304 (1.432–7.621)	0.01	2.757 (1.103–6.892)	0.03
Unemployed	142	7 (4.9)	0.052 (0.452–1.251)	0.00	0.091 (0.0514–1.322)	0.41
Civil servants	5	0 (0.0)	a	a	a	a
**Location**						
Sub-urban	305	28 (9.2)	2.004 (1.032–3.889)	0.04	1.993 (1.022–3.889)	0.05
Rural	95	16 (16.8)	a	a	a	a
**HIV**						
Positive	200	29 (14.5)	2.092 (1.084–4.035)	0.03	1.667 (0.793–3.505)	0.05
Negative	200	15 (7.5)	a	a	a	a
**Level of education**						
None	7	1 (14.3)	10.500 (0.580–89.98)	0.11	6.753 (0.362–126.067)	0.20
Primary	259	35 (13.5)	9.844 (1.322–73.27)	0.03	8.484 (1.131–63.622)	0.04
Secondary	70	7 (10.0)	7.000 (0.837–58.56)	0.07	5.199 (0.607–44.505)	0.13
Tertiary	64	1 (1.6)	a	a	a	a
**Number per house**						
1–4	209	21 (10.0)	1.628 (0.338–7.845)	0.54	1.503 (0.308–7.334)	0.61
5–9	178	21 (11.8)	1.359 (0.282–6.560)	0.70	1.259 (0.258–6.151)	0.78
10–14	13	2 (15.4)	a	a	a	a
**Source of water**						
Portable tap water	340	30 (8.8)	3.145 (1.552–6.371)	0.01	3.261 (1.595–6.668)	0.02
Other sources	40	14 (23.3)	a	a	a	a
**Method of faeces disposal**						
Pit toilet	320	29 (9.1)	2.316 (1.174–4.566)	0.02	2.346 (1.182–4.655)	0.03
Water system	80	15 (18.8)	a	a	a	a
**Type of house**						
Mud thatched	175	17 (9.7)	1.267 (0.667–2.408)	0.47	1.285 (0.674–2.452)	0.45
Cemented	225	27 (12.0)	a	a	a	a
**Keep animals or pets**						
No	185	17 (9.2)	1.419 (0.747–2.696)	0.29	1.400 (0.734–2.669)	0.31
Yes	215	27 (12.6)	a	a	a	a

a = reference category.

## Discussion

In developing countries, intestinal parasitic infections remain a serious public health problem, especially in HIV-infected individuals. This cross-sectional study carried out in Tubah assessed the burden of intestinal parasites among HIV-positive and negative individuals. The study also attempted to investigate risk factors associated with parasitic infections.

Results obtained revealed that the overall prevalence of intestinal parasitic infections was 11% (Table 2). Compared to other studies, the low prevalence recorded in this study could be related to the location of the study area and the common practice of regular deworming of the population by the Government. People in suburban centers are usually educated and have access to community health talk such that they are aware of methods of transmission and prevention. This is similar to the work carried out by Nkoa et al.^15^ in Yaounde, Cameroon, but contrasts with the findings of other studies carried out in other parts of Cameroon^6,16^.

*Entamoeba histolytica* was the most prevalent protozoan parasite (Fig. 2). This could be attributed to the very resistant cystic forms of the parasite which are transmitted by individuals seemingly looking healthy but who are carriers of the infective stage of the parasite. Equally the low level of sanitation and quality of water in the study area may have been the reason for this. This is consistent with previous studies carried out in Abuja^17^ and in the Centre region, Cameroon^18^. The most frequently encountered helminths eggs were those of *A. lumbricoides, Hookworm* spp, *Taenia* spp., and *T. trichiura.* These helminths are geohelminthes and given that the main activity of people leaving in this area is farming, they are constantly being exposed. This finding is in line with the work of Yamssi et al.^19^ in Douala.

Furthermore, this study also revealed that the prevalence of intestinal parasites was higher in HIV-positive individuals than HIV negative individuals (Table 2). HIV infection compromises the immunity of the infected individual and thus makes them more prone to intestinal parasitic infections than HIV-negative individuals. A high prevalence of intestinal parasites among HIV-positive patients has also been reported in other studies both in Cameroon and out of Cameroon^15,18^.

The prevalence of parasitic multiple infections was higher in HIV positive patients than in HIV negative individuals and the most common multiple infections were *E. histolytica* + *A. lumbricoides*. Again this could be due to the sensitivity of diagnostic techniques, study participants’ immunity status, environmental hygiene, socioeconomic status, access to safe water supply, or other factors. Multiple infections of intestinal parasites with HIV are common in most tropical and sub-tropical countries and are associated with common risk factors such as rural residence, lack of education, promiscuous defecation, lack of potable water, etc. HIV infection is the most common immunodeficiency condition with the hallmark of depleting CD4+ T lymphocytes which are essential components of the cell-mediated immune system thus exposing HIV persons to possibly multiple intestinal parasitic infections. These findings are similar to those of Mbiandou et al.^18^ in the center region of Cameroon although are in contrast with the work done by Lehman et al.^16^ were the most common multiple infections were *Entamoeba coli* + *Cryptosporidium* spp. *E. coli* is a non-pathogenic parasite that lives is symbiosis with human as such they are not being treated medically. On the other hand, *Cryptosporidium* spp is transmitted in contaminated water and in Tubah sub division, most of the inhabitants uses community water which is not usually treated and is in short supply most of the time. This is supported by the fact that the participants that used water system toilets were more infected in this study as such possible water contamination. Secondly, the people of this area often rate animals as a source of living and it known that the transmission of *Cryptosporidium* spp can also by direct contact with infected animals or people.

Our results also indicated that the prevalence of intestinal parasites was significantly (*p* = 0.004) greater in patients with a viral load of > 1000 copies/mL compared to patients with a viral load ≤ 1000 copies/mL (Fig. 3). This could be explained by the fact that a high viral load will lead to lower CD4^+^ T cell counts that will eventually lead to weakened immunity. As such the host becomes unable to eliminate these pathogens which thrive and cause diseases. Therefore the more immunological suppressed an HIV-positive individual is; the more susceptible he/she is to opportunistic intestinal parasitic infections. This is similar to a study carried out by Miressa and Dufera^20^ in Ethiopia.

Several risk factors of intestinal parasites were identified within the study population. Participants aged > 65 years had the highest infection rate of intestinal parasites than all the other age groups. This might probably be due to a decreased immunity and a decrease in personal hygiene and sanitation thus exposing them to intestinal parasites. This is not in line with the work of Mbiandou et al.^18^ where the infection rate was higher in the age group of 29–45 years.

Results also revealed that those who were self-employed (farmers, bike riders, businesses, builders, etc.) had the highest risk of being infected with intestinal parasites. This could be explained by their regular feeding with food provided by street food vendors, irrespective of sanitary conditions. Other self-employed such as farmers were equally on top of the parasitic infection chart, probably due to their contact with the soil, which predisposes them to geohelminthes. These findings agree with the work carried out by Bissong et al.^6^ in Bamenda, Cameroon.

Rural duellers had 1.9 times more risk of having intestinal parasites than sub-urban duellers. This can probably be explained by the differences in behaviour between individuals from these settings, both in terms of awareness and hygiene, but also lack or insufficient resources for diagnostics and treatments, the relatively low financial resources, the impoverishment of these individuals, and decrease of their immunological status. There is also the availability of antihelminthic drugs in sub-urban areas, hence reducing the prevalence of these parasites. Similar findings were observed by Ntonifor et al.^21^ and Gedle et al.^22^.

Those who had a primary level of education were 8.5 times more at risk of having intestinal parasites than those with a tertiary level of education. Most of the study participants were primary school leavers who could not continue schooling due to poverty, lack of educational facilities, and probably the lack of schools in the study area due to the ongoing Anglophone crisis, thus causing them to become engaged in farming, business, cattle rearing, etc. With their limited knowledge of the transmission and prevention of intestinal parasites, these activities, therefore, expose them to infection. This observation is in line with that of Miressa and Dufera^20^ who showed that educational status was significantly related to the level of intestinal parasitic infections.

Most of the study participants depended on portable tap water as their source of water for house whole use which was very epileptic, so they often used other sources of water (e.g. wells, rivers, springs, and boreholes) to meet up with their demands. The use of water from different sources (not treated) might have contributed to the high prevalence of intestinal parasites in people depending on portable tap water. These observations are similar to those of Miressa and Dufera^20^ who identified the lack of safe water sources and usage as meaningfully connected with intestinal parasitic infections.

Method of stool disposal significantly (*p* = 0.013) affected the prevalence of intestinal parasitic infections. According to this study, those that used the water system toilets were 3.261 times more at risk of having intestinal parasites. Again this might be because they did not always have water to use, and so they mostly turned to using other sources such as bushes, streams, rivers, and pit toilets (if available) to dispose of faeces, thereby exposing themselves to intestinal parasites. Koumba et al.^23^ observed that the method of stool disposal had a significant effect on the prevalence of intestinal parasitic infections, but also remarked that those who used pit toilets harboured more intestinal parasitic infections.

## Conclusions

Considering that intestinal parasitosis was significantly higher in HIV positive individuals and the prevalence was significantly greater in patients with viral load of > 1000 copies/mL requires that diagnosis of intestinal parasitic infections should be routinely performed in HIV positive patients to improve the management and care given to them. Also, public health measures should continue to emphasize on the importance of good environmental and personal hygiene as well as provide and monitor the quality of drinking water which will go a long way to improve the quality of life in the inhabitants of this area.

## Data Availability

The datasets used and/or analyzed during this study are available from the corresponding author on reasonable request.

## Abbreviations

*A*: *Ascaris*
AIDS: Acquired immunodeficiency syndrome
ART: Antiretroviral treatment
*C.*: *Cryptosporidium*
*E*: *Entamoeba*
*G.*: *Giardia*
HIV: Human immunodeficiency virus
*N.*: *Necator*
SPSS: Statistical Package for the Social Sciences
RNA: Ribonucleic acid
*T.*: *Trichuris*
